# The frequency of involvement of head & neck sites in referred otalgia – An experience at a tertiary care hospital

**DOI:** 10.12669/pjms.35.4.236

**Published:** 2019

**Authors:** Khurshid Anwar, Shehreyar Khan, Isteraj Shahabi, Zenab Berches Niazi

**Affiliations:** 1 Khurshid Anwar, Associate Professor, Department of ENT and Head &Neck Surgery, Hayatabad Medical Complex, Peshawar, Pakistan; 2 Shehreyar Khan, Specialist Registrar, Department of ENT and Head &Neck Surgery, Hayatabad Medical Complex, Peshawar, Pakistan; 3 Isteraj Shahabi, Professor & Head, Department of ENT and Head &Neck Surgery, Hayatabad Medical Complex, Peshawar, Pakistan; 4 Zenab Berches Niazi, Post Graduate Trainee, Prosthodontics, Khyber College of Dentistry, Peshawar, Pakistan

**Keywords:** Earache, Otalgia, Referred Pain, Tonsillitis, Laryngeal neoplasms

## Abstract

**Objective:**

To determine the frequency of involvement of distant head & neck sites which share sensory innervations with the ear in referred otalgia.

**Methods:**

This prospective study was conducted in the Department of ENT at Hayatabad Medical Complex, Peshawar, during the period from July 1, 2017 to December 31, 2017.*Non probability convenience sampling technique was used to include patients in the study. Patients with true ‘otogenic pain’ as determined on clinical examination or imaging studies were excluded from analysis. Only those who presented with referred ear ache were included in the study to determine the frequency of involvement of distant head & neck sites in ‘referred otalgia’. Otalgia was designated as “Unknown Origin” when the ear and distant sites too were found normal.

**Results:**

Out of a total of 150 patients, there were 81(54%) males and 69(46%) females. The ages ranged from 5 to 66 years with a mean age of 29.15 years. The commonest age groups involved were 21-35 years and 36-55 years. Referred otalgia of tonsillar origin was found in 47(31.3%) of patients followed by that of dental origin in 35(23.3%). Otalgia due to pharyngitis, rhinosinusitis and cervical origin was 24(16%), 8(5.3%) and 6(4%) respectively. Otalgia due to temporomandibular joint was noted in 12(8%) of females and 3(2%) of males & that of “unknown origin” affected 5(3.33%) of females and 2(1.3%) of males.

**Conclusions:**

The ear should be examined in detail in patients presenting with earache to look for indigenous pathology. In the presence of a ‘Normal Ear’, it is important to examine the tonsils, teeth, pharynx and the nose & paranasal sinuses as the possible sites of origin of earache.

## INTRODUCTION

Earache is a common presentation in the ENT outpatient clinic. In most cases the cause lies in the ear itself. However, it can pose a diagnostic problem when examination reveals no pathology in the ear. Earache may be otogenic when the cause lies in the ear or non-otogenic when the origin is at sites other than the ear.^1^ This latter entity is commonly known as “Referred Otalgia”. The severity of pain varies among these patients but is severe enough to warrant treatment. The aetiology of referred otalgia is diverse and pain may arise from head neck sites with which the ear shares its sensory innervations.

An understanding of the innervation of the head and neck region plays a key role in making a correct diagnosis of referred otalgia. The ear shares its sensory innervation through the 5^th^, 7^th^, 9^th^ & 10^th^ cranial nerves and C2 & C3 spinal nerves. The mechanism of referred otalgia is poorly understood. However, the most accepted theory is the convergence projection theory which states that multiple nerves converge onto a single shared neural pathway with the CNS unable to differentiate the origin of stimulation.^2^,^3^

Irritative lesions in the areas supplied by these nerves such as the neck, nose & paranasal sinuses, nasopharynx, oral cavity, oropharynx, hypopharynx and larynx may also cause pain in the ear. The temporomandibular joint causes earache both by its contiguity to the ear and shared sensory supply. It is therefore, of immense importance to look for any pathology in the areas supplied by these nerves.

The origins of referred otalgia may include the cranial cavity to thorax but dental diseases, tonsillitis, temporomandibular joint and cervical spine disorders are the commonest sites.^4^ Referred otalgia may be the first symptom of a head and neck malignancy. Patients with risk factors for upper aero digestive tract malignancies and normal ear examination require further evaluation.^5^,^6^

In otalgia with negative findings on otoscopic and CT examination of the temporal bone, it is imperative that distant sources within the head & neck be evaluated. This may include inspection with or without the use of flexible endoscopes and palpation of the accessible areas. CT and MR imaging are excellent tools to investigate further regions of the head and neck that are not easily accessible to physical examination.^7^

The patients presenting with pain in the ear receive little attention especially when the ear is found to be normal on otoscopic examination. The attending clinician needs to be aware of the likelihood and predilection of various non-otogenic causes of earache. As little work has been done in this regard in our settings, this study aims to share our experience with clinicians. Knowing the frequency of involvement of various distant primary sites that share sensory innervation with the ear will help the attending physician to prioritize his examination. Moreover, the fact that presence of early malignancies at the primary sites with referred earache as the only symptoms, emphasizes referred otalgia as a significant and an important entity.

## METHODS

This prospective and descriptive study was conducted at the Departments of ENT and Head & Neck Surgery, Hayatabad Medical Complex, Peshawar from July 1, 2017 to December 31, 2017. Non-probability convenience sampling technique was used to induct patients in the study. Ethical approval for conducting the study was obtained from institutional ethical committee. All patients over five years of age, both male and female, and presenting with ear ache were selected. Patients having aural pathology to account for pain, had previous surgery on the affected ear or having hemi facial pain or vague sensation of aural fullness were excluded from analysis. A total of 450 patients presenting with earache were examined. The procedure was explained and Informed consent obtained. Detailed history taking & clinical examination ensued. Laboratory investigations, imaging studies and endoscopic examinations were carried out as and when indicated. Final diagnosis of referred otalgia was made on positive findings in the other head & neck sites that share sensory innervation with the ear in conjunction with a “Normal on Examination” affected ear. The earache was designated as otalgia of “Unknown Origin” when the distant sites too were found normal on clinical examination and imaging studies. Only 150 patients were diagnosed as having referred otalgia.

### Statistical analysis

The data was recorded on a proforma and the descriptive statistics were analyzed to determine frequencies for variables like gender, age and type of otalgia. Percentages were calculated for the number of times a distant site was implicated in referred otalgia in relation to various age groups and gender. Chi-Square test was applied to determine the significance of involvement of distant sites with respect to the variables; age groups and gender of patients. The analysis was carried out using SPSS 16 for windows.

## RESULTS

A total of 450 patients presenting with earache were examined. Referred otalgia was diagnosed in 150 (33.33%) patients and the rest were excluded from analysis. The ages of the patients ranged from 5 to 66 years with a mean age of 29.15 years and a standard deviation of +/- 15.0. The male to female ratio stood at 1.2:1 with 81(54%) males and 69(46%) females. The frequency of involvement of various age groups has been shown in Table-I. Referred otalgia of tonsillar origin was noted in 47(31.3%) of patients and was the most frequent findings. In 7(4.7%) of patients no lesions were found in the distant head & neck sites to account for the ear ache. The frequency with which the various head and neck sites were involved in referred otalgia has been given in Table-II. Otalgia due to temporomandibular joint disease, hypopharyngeal carcinoma and that of “unknown origin” was frequently seen in females than in males (Table-III). Chi-Square test revealed that statistically this difference has been insignificant (p-value >0.05). Referred pain due to tonsillar and dental causes was commonly found in the younger age groups as compared to that of cervical, hypopharyngeal and laryngeal origins (Table-IV). Statistically, however, this predilection for involvement of various age groups was found to be non significant (p-value >0.05).

**Table I:** Age groups of the patients.

Age Groups		Frequency	Percent	Cumulative Percent
Valid	5-10	14	9.3	9.3
	11-20	39	26.0	35.3
	21-35	52	34.7	70.0
	36-55	36	24.0	94.0
	56-70	9	6.0	100.0

	Total	150	100.0	

**Table II:** Frequency of origin of otalgia.

	Origin of otalgia	Frequency	Percent	Valid Percent	Cumulative Percent
Valid	Tonsillar origin	47	31.3	31.3	31.3
	Dental origin	35	23.3	23.3	54.7
	Pharyngitis	24	16.0	16.0	70.7
	TMJ origin	15	10.0	10.0	80.7
	Nose & sinus origin	8	5.3	5.3	86.0
	Cervical origin	6	4.0	4.0	90.0
	Hypopharyngeal Carcinoma	5	3.3	3.3	93.3
	Laryngeal carcinoma	3	2.0	2.0	95.3
	Unknown origin	7	4.7	4.7	100.0

	Total	150	100.0	100.0	

**Table III:** Origin of otalgia in relation to gender of the patients.

Origin of otalgia	Gender of patients		Total

	Male	Female	
Tonsillar origin	27	20	47
Dental origin	21	14	35
Pharyngitis	16	8	24
TMJ origin	3	12	15
Nose & sinus origin	5	3	8
Cervical origin	3	3	6
Hypopharyngeal Carcinoma	1	4	5
Laryngeal carcinoma	3	0	3
Unknown origin	2	5	7

Total	81	69	150

**Table IV:** Origin of otalgia in relation to age groups of patients.

Origin of otalgia	Age Groups					Total

	5-10	11-20	21-35	36-55	56-70	
Tonsillar origin	9	19	16	3	0	47
Dental origin	5	6	12	8	4	35
Pharyngitis	0	6	11	7	0	24
TMJ origin	0	4	4	7	0	15
Nose & sinus origin	0	4	4	0	0	8
Cervical origin	0	0	0	5	1	6
HypopharyngealCarcinoma	0	0	1	3	1	5
Laryngeal carcinoma	0	0	0	0	3	3
Unknown origin	0	0	4	3	0	7

Total	14	39	52	36	9	150

## DISCUSSION

Primary ear pain is relatively simple to diagnose. However, the ear is not always the source in ear ache. It becomes a diagnostic dilemma to identify the source of pain when the ear is found normal on examination. A number of nerves that supply other head and neck structures also supply the ear. A variety of pathological conditions ranging from inflammation to neoplasia can cause irritation of these sensory nerves and cause pain radiating to the ear by virtue of their shared sensory innervation.

The national literature on this particular subject is scarce and majority of the studies are focused on the aetiology of primary otogenic pain. Literature search from other countries on referred otalgia highlights various aspects of the problem.^8^-^10^ Modern gadgets for investigations have favorably aided the clinicians to make correct diagnoses. The association of head and neck malignancies with referred pain to the ear warrants a careful attention towards an apparently a minor symptom.

Pain in the ear, for most part, is caused by primary pathology in the ear. In United States, a retrospective analysis of Nationwide Emergency Department Sample (NEDS) Database for the period from 2009 to 2011 by Kozen et al, 8611282(2.21%) of all emergency department visits were paid for otologic complaints. It was observed that most commonly diagnosed conditions were otitis media not otherwise specified (NOS) (60.6%), infected otitis externa NOS (11.8%) and otalgia NOS (6.8%).^11^

In our study, tonsillar and dental causes were found to be the commonest entities, cervical spine and TMJ disorders were responsible for a small proportion of referred otalgia. This is in contrast to a national study involving 216 patients conducted at the Zia-ud-Din Hospital Karachi, by Baig S et al. the quoted study results suggested frequency of otalgia in 55.1% (Ear infections), 29.2% (TMJ) and 15.7% due to neck pain.^12^

Quite a number of studies in the literature regard dental causes to be the commonest aetiology of referred otalgia.^13^,^14^ However such findings may differ widely. More than one aetiology may be present simultaneously and the earache may be unilateral or bilateral. A recent Turkish study involving 224 patients, Mazlumoglu MR and colleagues identified cervical spine disease (49.1%) and temporomandibular joint disease (32.1%) as the leading causes of referred ear ache. They found dental pathologies to account for in 21.9% of their patients. Chronic sinusitis was responsible for 9.4% of patients with referred otalgia. They also found more than one pathology in 31.3% of their patients as a cause for non otogenic pain.^15^

In the US, Jaber et al studied 123 patients presenting with referred otalgia. The mean age of the patients was 63.5 years. They found that 84% of their patients with referred ear pain had some form of cervical spine pathology. They concluded that cervical spine degenerative disorders constitute important causes of referred otalgia in the elderly.^16^ In a Korean study of 294 patients with otalgia, the frequency of primary otalgia was found to be higher in children than in adults and in men than in women. This study supports our findings that referred otalgia was more likely to occur in adults in general and in women in particular. Similarly, otalgia of “unknown origin” was found to be more common in females than in males.^17^ In a study at Mashhad University in Iran, Taziki MH found that referred otalgia of dental origin was found in 62.8% patients. We found otalgia of tonsillar origin to be the commonest (31.3%) and otalgia of dental origin was found only in 23.3% of the patients.^13^

Our findings are also supported by a similar study in Iraq. Taboo ZA and Burra MF found that referred otalgia was more common in adults than in children and that temporomandibular joint disorders were predominantly responsible for referred pain in females.^18^ We also noted 3.3% of patients with hypopharyngeal carcinoma and 2% of cases with laryngeal carcinoma having pain in the ear. In Indian study conducted Sumitha R and Joseph NA, 2% of the subjects with hypopharyngeal carcinoma had earache as the presenting symptom.^19^

Neuralgias are also implicated in the causation of referred earache. These are a group of disorders causing severe pain in the distribution of the affected nerve without producing neurological deficit. Diagnosis is made by excluding anatomic basis for the patient’s symptoms.^8^ We encountered 4.7% of our patients in whom no cause for the earache could be found either in the ear or distant head neck sites having common sensory innervation with the ear. These patients lacked the “lancinating” character of pain typical of neuralgia and rather described their pain was intermittent and disturbing in nature.

## CONCLUSION

In patients who complain of earache, quite a number of patients have no pathology in the ear. In the presence of a ‘Normal Ear’, it is important to examine the tonsils, teeth, pharynx, and the nose & paranasal sinuses as the likely sites of origin of earache. With malignancy as a possible pathology at these sites, a thorough and careful examination is advisable.

### Author`s Contribution

**KA:** Principal author. Conception of design, acquisition of data, analysis of data, critical analysis of content, drafting the article.

**SK:** Acquisition of data, analysis of data, drafting the article.

**IS:** Acquisition of data, analysis of data, critical analysis of content, drafting the article and final approval.

**ZBN:** Acquisition of data, analysis of data & content, drafting the article.
